# Predictive factors of malignancy in pediatric patients with thyroid nodules and performance of the Italian classification (SIAPEC 2014) in the outcome of the cytological FNA categories

**DOI:** 10.1007/s12020-021-02784-0

**Published:** 2021-06-14

**Authors:** Gerdi Tuli, Jessica Munarin, Erica Agosto, Patrizia Matarazzo, Francesco Quaglino, Alberto Mormile, Luisa de Sanctis

**Affiliations:** 1 Department of Pediatric Endocrinology, Regina Margherita Children’s Hospital, Turin, Italy; 2 Department of Public Health and Pediatrics, University of Turin, Turin, Italy; 3 Department of General Surgery, “Maria Vittoria” Hospital ASL Città di Torino, Turin, Italy; 4 Endocrinology, Diabetes and Metabolism Department and Center for Thyroid Diseases, Ordine Mauriziano Hospital, Turin, Italy

**Keywords:** Thyroid nodule, Pediatric, FNAB, Cytological category, Outcome

## Abstract

**Purpose:**

The rate of malignancy (ROM) among pediatric studies using the Bethesda System is 39.5% and 41.5% for atypia of undetermined significance/follicular lesion of undetermined significance and for suspected follicular neoplasm, respectively. Data reported on the basis of Bethesda System showed lower ROM in adults with indeterminate nodules (30.5 and 28.9% respectively). Studies on adults based on the Italian Society of Anatomic Pathology and Cytology (SIAPEC) classification, report ROM of 14.2% for TIR3a and 44.6% for TIR3b category, showing greater sensitivity in detecting malignancy. To date, no performance data are available about SIAPEC classification in pediatric age.

**Methods:**

Retrospective data were collected from 200 pediatric subjects with thyroid nodules in the period 2000–2020.

**Results:**

The distribution of cytological categories after fine needle aspiration biopsy (FNAB) was 7 TIR1, 4 TIR1c, 22 TIR2, 14 TIR3a, 9 TIR3b, 3 TIR4, and 16 TIR5. The surgical approach was performed in 40/200 subjects, with total ROM of 65% (0% for TIR1-TIR3a, 77.8% for TIR3b, and 100% for TIR4–TIR5). Total FNAB accuracy was 95%, while the sensibility and specificity were 92.3 and 92.6%, respectively.

**Conclusions:**

The reported data seem to confirm a greater sensitivity of SIAPEC classification to identify malignancy within the indeterminate category also in pediatric age and not only in adulthood. This finding may orient clinicians toward clinical follow-up for the indeterminate TIR3a group and toward surgical approach with total thyroidectomy in the indeterminate TIR3b group, although this indication should be confirmed in further national multicenter studies including larger cohorts.

## Introduction

Nodular thyroid disease in pediatric age has a lower prevalence (0.2–5.1%) than in adulthood (1–10%) [1–3], but the main difference between pediatric and adult age lies in the rate of malignancy (ROM) (16–26% vs 5–10%) [4, 5]. Differentiated thyroid cancer (DTC), which occurs mainly in adolescence and in females, accounts for 30% of endocrine tumors, which in turn represent 4–5% of all childhood neoplasms [6, 7]. Among DTCs, papillary thyroid cancer (PTC) is the most frequent histological type (90%), while follicular thyroid cancer (FTC) and medullary thyroid cancer (MTC) are rare [8–10]. Thyroid disease, radiation exposure, previous neoplasms, family history, young age, male gender, and genetic predisposition are considered to be the most important risk factors for developing thyroid cancer [1–20].

Anamnestic and clinical data, thyroid hormone profile, and ultrasound evaluation are critical for the initial assessment [11–21]. Ultrasound features suspected for malignancy include nodule size >1 cm, hypoechogenicity, intranodal vascularity or microcalcifications, irregular borders, and neck lymph nodes [5, 21–28]. Once suspicious ultrasound features are present, fine needle aspiration biopsy (FNAB) is required to determine the cytological category. The most widely used cytological classifications are the Bethesda System for Reporting Thyroid Cytopathology (BSRTC) and the British Thyroid Association (BTA) Guidance on the reporting thyroid cytology; in Italy, the Italian Society of Anatomic Pathology and Cytology (SIAPEC) in 2014 reviewed the cytological categories, indicating a five-tiered classification and subdividing the ‘indeterminate for malignancy’ class into the ‘TIR3a’ low-risk indeterminate lesion (LRIL) and the ‘TIR3b’ high-risk indeterminate lesion (HRIL) due to the follicular features of the neoplasia (Table 1) [29–31].

**Table 1:** SIAPEC, Bethesda and BTA cytological categories, rate of malignancy for each category and suggested management approach

SIAPEC			BSRTC			BTA		
Category	ROM	Suggested approach	Category	ROM	Suggested approach	Category	ROM	Suggested approach
TIR1/TIR1c ND/Cystic Undiagnostic or cystic but not colloidal	Not defined	Repeat FNAB	I ND	5–10%	Repeat FNAB	THY1/THY1c ND/Cystic	4%	Repeat FNAB
TIR2 NM/B Colloidal or hyperplastic cystic lesions	<3%	Follow-up	II B	0–3%	Follow-up	THY2/THY2c B	1.4%	Follow-up
TIR3a LRIL High cellularity, low colloidal content, numerous follicular structures but not FN	<10%	Repeat FNAB Follow-up	III AUS/FLUS	10–30%	Repeat FNAB Follow-up or lobectomy	THY3a Atypia/ND	17%	Repeat FNAB Follow-up
TIR3b HRIL High cellularity, monotone disposition in microfollicular/trabecular structures, and low/absent colloidal content: FN	15–30%	Surgery	IV SFN	25–40%	Molecular testing, lobectomy	THY3b FN	Up to 40%	Surgery
TIR4 SM	60–80%	Surgery	V SM	50–75%	Lobectomy or near-total surgery	THY4 SM	Up to 68%	Surgery
TIR5 M	95%	Surgery	VI M	97–99%	Lobectomy or near-total surgery	THY5 M	Up to 100%	Surgery

*ROM* rate of malignancy, *ND* not diagnostic, *NM* not malignant, *B* Benign, *LRIL* low-risk indeterminate lesion, *HRIL* high risk indeterminate lesion, *SM* suspicious for malignancy, *M* malignant, *AUS/FLUS* atypia of undetermined significance/follicular lesion of undetermined significance, *SFN* suspicious follicular neoplasm, *FN* follicular neoplasm

In adulthood, each category has a ROM that varies according to the classification system used; to date few data on pediatric age are available. All classification systems include categories for non-diagnostic cytology samples, benign lesions, and malignant lesions, but differ in the terminology of borderline lesions. The BSRTC indicates two distinct categories for borderline lesions: “Atypia of undetermined significance (AUS)/follicular lesion of undetermined significance (FLUS)” and “suspicious follicular neoplasm (SFN)” (III–IV categories). The British system uses the “ThyIII” category for all borderline cases, differentiating in “ThyIIIa” the possible neoplasm with atypia and in “ThyIIIf” the possible follicular neoplasm, similar to the SIAPEC classification.

For the adult population, some Italian authors have studied national cohorts affected by thyroid nodules and reported significantly higher sensitivity of the Italian system than the Bethesda one, validating the TIR3a/TIR3b subclassification for the individuation of benign nodules and malignant tumors, with a ROM of 4–20.8% and 28–60.3%, respectively [32–43]. Pediatric studies based on the BSRTC system indicate a higher ROM for indeterminate nodules (39.5% for AUS/FLUS category [44] and 41.5% for the SFN category) compared to adults (30.5 and 28.9% respectively), with no substantial differences in ROM between the two indeterminate categories in both pediatric and adult age [45–58].

To our knowledge, according to the SIAPEC classification, no pediatric ROM data are currently available for each cytological category. The aim of this retrospective study is to represent the clinical features of pediatric patients with thyroid nodules and to analyze the ROM and the outcomes between the indeterminate cytological categories TIR3a and TIR3b.

## Materials and methods

This retrospective study included all subjects younger than 18 years old with thyroid nodules greater than 0.5 cm referred to the Tertiary Center for Pediatric Endocrinology of Regina Margherita Children’s Hospital in Turin in the period 2000–2020. After approval by the Institute’s Ethical Committee, clinical, laboratory, and radiographic data were collected from electronic medical records. Age, sex, familiarity, and previous radiation exposure were documented. Patients were classified as euthyroid, hypothyroid, or hyperthyroid based on serum levels of thyrotropin, free thyroxine, and free triiodothyronine. Serum calcitonin was obtained in all patients with a history of MTC or nodules larger than 1 cm. All patients underwent thyroid ultrasound evaluation, which included the nodule diameter and the echoic pattern, classified as anechoic, hypoechoic, isoechoic, hyperechoic, or mixed. Lymph node changes, such as rounded bulging shape, irregular margins, increased size, absence of echogenic hilum, heterogeneous echoic pattern, presence of calcifications or cystic areas, and irregular vascularity were recorded. Technetium Tc^99^ scintiscan was performed only in hyperthyroid subjects. A cytological sample was obtained through ultrasound-guided FNAB in case of nodules greater than 1 cm or in case of suspicious features of malignancy at ultrasound evaluation. The cytological category was defined according to the SIAPEC classification. In its 2014 revision, the previous category of indeterminate cytology (TIR3) was further divided into TIR3A (low risk) and TIR3B (high risk due to features of follicular neoplasm). In subjects undergoing surgery for lobectomy or total thyroidectomy, histological specimens were also obtained. All specimens were evaluated by a single pathologist and have been reviewed for the study and the previous TIR3 category was reclassified in the new TIR3a and TIR3b categories by the same pathologist.

Statistical analysis and graphs construction were performed using GraphPad 7 (GraphPad Software, La Jolla, CA, USA). The sensitivity of FNAB (number of true positives divided by the sum of true positives and false negatives), its specificity (number of true negatives divided by the sum of true negatives and false positives), and diagnostic accuracy (sum of true positives and true negatives divided by the number of the sample) were calculated based on the results of patients undergoing both FNAB and surgery. Differences between groups were established by *t* test to compare mean values of continuous variables. For sample sizes smaller than 15, a Fisher exact test was used. The calculations were considered statistically significant when the *P* value was less than 0.05.

## Results

We collected the clinical, laboratory and ultrasound retrospective data of 200 subjects (119 females and 81 males, 174 benign and 26 malignant nodules) younger than 18 years old with thyroid nodules (Table 2). The overall ROM observed was 13%, which rises up to 26% if nodules with a diameter greater than 1 cm were considered. The mean age at diagnosis was 11.6 years old (range 2–18), with a mean duration of follow-up of 8.6 years. The female-to-male ratio was 1.47, dropping to 1.27 by considering malignant nodules.

**Table 2:** Clinical, biochemical, and US features of all subjects (*N* = 200) with thyroid nodule

Clinical, biochemical, and US features	All (*n* = 200)	Benign (*n* = 174)	Malignant (*n* = 26)	*p*
Age at diagnosis (years)	12 (2–18)	12 (2–18)	12.9 (7–17.1)	0.22
Gender (M/F)				
M	81	70	11	0.65
F	119	104	15	
Thyroid disease familiarity	108	96	12	0.66
Radiation exposure	28	22	6	0.1
TSH (mcUI/ml)	2.01 (0.1–5.3)	1.94 (0.1–4.9)	2.56 (0.8–5.3)	**0.01**
fT4 (pg/ml)	11.5 (1.05–31.4)	11.5 (1.05–31.4)	12 (7.3–15.3)	0.55
fT3 (pg/ml)	4.05 (2.3–5.8)	4 (3.06–5.8)	4.09 (2.3–4.6)	0.18
Thyroid antibodies positivity	69	53	16	0.8
Nodule localization				
Left lobe	91	86	5	**0.01**
Right lobe	92	75	17	
Bilateral	17	13	4	
Major nodule diameter (mm)	9 (8–60)	8 (8–10)	24 (7–60)	**<0.0001**
Echoic pattern				
Hypoechoic	121	101	20	0.24
Hyperechoic	14	14	0	
Isoechoic	20	18	2	
Anechoic	17	17	0	
Mixed	28	24	4	
Intranodal vascularity	61	46	15	**0.003**
Intranodal calcifications	23	15	8	**0.009**
Lymph node involvement	11	1	10	**<0.0001**

The bold values indicates the statistically significant results

Familial anamnesis for autoimmune thyroid disease was positive in 108/200 (53.9%), while 69/200 subjects (34.6%) had autoimmune thyroid disease. Radiation exposure due to previously radiotherapy-treated cancer was present in 28/200 (14.1%) of subjects. No risk factors were found in 35/200 (17.3%) cases. Regarding the risk factors investigated, no difference was observed among benign and malignant nodules.

Laboratory data showed mean TSH levels of 2.01 mcUI/ml (0.1–5.3), mean fT4 levels of 11.5 pg/ml (1.05–31.4), and mean fT3 levels of 4.09 pg/ml (2.3–4.6). TSH levels were significantly lower in subjects with a benign nodule than in subjects with a malignant nodule, being 1.94 mcUI/ml (0.1–4.9) vs 2.56 mcUI/ml (0.86–5.3), respectively (*p* = 0.01)*.* No differences were observed for fT4 and fT3 levels or for the presence of anti-peroxidase and anti-thyroglobulin antibodies.

Ultrasound evaluation showed the localization of the nodule in the right lobe in 92 subjects (46%), the involvement of the left lobe in 91 subjects (45.5%), and the bilateral localization in 17 (8.5%). Bilateral and right lobe involvement was associated with a higher malignancy rate than left lobe localization (23.6% vs 18.5% vs 5.5% malignancy rate, respectively, *p* = 0.01). Intranodal vascularization was present in 61/200 (30.4%), while intranodal calcification in 23/200 (11.5%); both were associated with higher malignancy rate (*p* = 0.003 and *p* = 0.009, respectively), as well as lymph node involvement (*p* < 0.0001) A larger nodule diameter was significantly more present in the malignant nodule than in the benign nodule group (mean diameter 24 mm vs 8 mm, respectively, *p* ≤ 0.001). The hypoechoic pattern was the most frequently observed ultrasound feature for both groups, with no correlation with the ROM.

When considering the indeterminate cytology category, no differences were observed in terms of age, gender, family history, genetic mutations, radiation exposure, thyroid hormone profile and ultrasound pattern, as well as the presence of intranodal calcifications or vascularization (Table 3). Localization of the nodule in the right lobe and larger diameter were associated with a higher ROM (*p* = 0.01 and *p* < 0.003, respectively), as well as lymph node involvement (*p* < 0.0001).

**Table 3:** Clinical, laboratory, and US features of subjects with the cytological category of indeterminate thyroid nodule

Clinical, biochemical, and US features	TIR3a (*n* = 14)	TIR3b (*n* = 9)	*p*
Age at diagnosis (years)	13.25 (6–17.5)	13 (6–15)	0.66
Gender (M/F)			
M	7	5	0.62
F	7	4	
Thyroid disease familiarity	0	2	0.99
Radiation exposure	3	1	0.22
TSH (mcUI/ml)	1.95 (0.22–4.9)	1.6 (1.09–3.34)	0.6
fT4 (pg/ml)	12.1 (9.1–13.8)	12.7 (10.5–14.9)	0.2
fT3 (pg/ml)	4.2 (3.2–5.8)	4.3 (2.3–4.6)	0.18
Thyroid antibodies positivity	0	2	0.15
Nodule localization			
Left lobe	12	2	**0.01**
Right lobe	2	6	
Bilateral	0	1	
Major nodule diameter (mm)	8 (5–33)	25 (20–60)	**<0.003**
Echoic pattern			
Hypoechoic	5	5	0.4
Hyperechoic	2	0	
Isoechoic	3	0	
Anechoic	0	0	
Mixed	4	4	
Intranodal vascularity	7	6	0.94
Intranodal calcifications	3	2	0.99
Lymph node involvement	0	5	**<0.0001**

The bold values indicates the statistically significant results

Of the 174 subjects with benign nodules, 125 were evaluated by periodical clinical and ultrasound check, FNAB was performed in 49 subjects, whereas 14 underwent to surgery.

FNAB was performed on the basis of nodule size and ultrasound feature in 75/200 (37.5%) of subjects, including 7 TIR1 (9.3%), 4 TIR1c (5.3%), 22 TIR2 (29.3%), 14 TIR3a (18.7%), 9 TIR3b (12%), 3 TIR4 (4%), and 16 TIR5 (21.4%) (Table 4).

**Table 4:** Cytological, histological data, malignancy rate, and FNAB accuracy for each cytological category

SIAPEC category	Nr	Surgery	Outcome	ROM	FNAB accuracy
TIR1	7 (9.3%)	–	Ultrasound and FNAB follow-up,	0%	–
			3 TIR2 at FNAB repetition		
TIR1C	4 (5.3%)	1	Ultrasound follow-up;	0%	100%
			1 ST for cystic nodule and dysphagia		
TIR2	22 (29.3%)	5	Ultrasound follow-up;	0%	100%
			5 TT for important MNS		
TIR3a	14 (18.7%)	1 TT	5 ST with adenoma histological diagnosis	0%	100%
		5 ST	1 TT with MNS histological diagnosis		
			8 patients still undergoing ultrasound/FNAB follow-up		
TIR3b	9 (12%)	7 TT	All subjects underwent surgery;	77.8%	77.8%
		2 ST	7 TT of which 5 PTC histological diagnosis, 1 PMC, and 1 FTC		
			2 ST with NS histological diagnosis		
TIR4	3 (4%)	3 TT	All subjects underwent surgery;	100%	100%
			PTC histological diagnosis in all subjects		
TIR5	16 (21.4%)	16 TT	All subjects underwent surgery;	100%	100%
			PTC histological diagnosis in all subjects		
Total	75	40		65%	95%

*ROM* rate of malignancy, *ST* subtotal thyroidectomy, *TT* total thyroidectomy, *MNS* multinodular struma, *NS* nodular struma, *PMC* papillary micro carcinoma, *PTC* papillary thyroid carcinoma, *FTC* follicular thyroid carcinoma

Surgery was performed in 40/200 (20%), with a total malignancy rate of 65% (0% for the TIR1-TIR3a, 77.8% for the TIR3b and 100% for the TIR4–TIR5 categories).

Of the 14 subjects with cytological category TIR3a, 6 underwent surgery, 5 by lobectomy, with a histological diagnosis of thyroid adenoma, and 1 by total thyroidectomy, with histological diagnosis of multinodular struma. The mean duration of follow-up was 4.6 years and FNAB was repeated in the remaining 8 subjects every 12–18 months, with confirmation of the cytological category.

All subjects with indeterminate TIR3b category underwent surgery; 7 of them underwent total thyroidectomy, with final diagnosis of PTC in 5 cases, FTC in 1 case, and papillary microcarcinoma in 1 case, while the remaining 2 subjects underwent lobectomy, with final diagnosis of multinodular struma.

All subjects with cytological categories TIR4–TIR5 underwent total thyroidectomy and histological analysis confirmed a PTC in all cases.

The total accuracy of FNAB was 95%. For the benign low-risk categories TIR1–TIR2 and the malignant categories TIR4–TIR5 the FNAB showed 100% accuracy, whereas for indeterminate categories TIR3a and TIR3b accuracy was 100 and 77.8%, respectively. The sensitivity and specificity of FNAB for all categories were 92.3 and 92.6%, respectively.

## Discussion

Nodular thyroid disease in pediatric age is a rare condition with prevalence of 0.2–5.1%, much lower than the adult population (1–10%); however, children and adolescents are known to have a higher malignancy rate (12.5–50%), with an average rate of 20–25%, much higher than the adult population of 5–10% [1–7]. *In our cohort*, gender, previous thyroid disease and radiation exposure were not related to malignancy. As previously shown by several authors, TSH levels were directly related to malignancy [20, 45–48]. Larger nodule size, presence of intranodal calcification or vascularity, and lymph node involvement were also associated with malignancy, as previously reported [1–3, 22–25]. Other authors have also indicated that hypoechogenicity and suspected neck lymph nodes are related to malignant neoplasms [5, 21–28]. In our cohort, no difference in the echoic pattern was observed, but the bilateral localization of the nodule and its presence in the right lobe were significantly associated with a higher ROM, a finding to our knowledge so far not yet reported in *pediatric age* in the literature.

Nodules larger than 1 cm or with ultrasound features suspected of malignancy are subjected to echo-guided FNAB. The 2014 Consensus Statement of the SIAPEC revised the cytological classification by diving the previous indeteminate category TIR3 in two sub-categories: TIR3a, if the lesion is considered low risk, and TIR3b, if considered at high risk. Clinical follow-up and eventual repetition of FNAB is the recommended approach for TIR3a nodules, while surgery for TIR3b nodules. It is noteworthy that, before 2014, all TIR3 classified nodules were treated by surgical exeresis; the new classification therefore allows to avoid the excessive surgical approach in the nodules classified TIR3a. The BSRTC and BTA Guidance on the reporting thyroid cytology, which are the most worldwide used cytological classifications, also consider two categories of indeterminate lesion: AUS/FLUS and “SFN” (III-IV categories), the former; ThyIIIa, for possible neoplasm with atypia, and ThyIIIf, for possible neoplasm suggesting follicular neoplasm, respectively, the second. The main difference of the three cytologic classifications is in the indeterminate group. The interpretation of “atypia” features is different for the SIAPEC classification, which includes such nodules in TIRb category, while BTA and BSRTC include some of them in the THY3a and category III, respectively.

To our knowledge, this is the first study that analyzes the performance of the SIAPEC cytology classification in pediatric age. We compared the data of our study with the results of similar studies on Italian adult cohorts and the main studies on pediatric cohorts based on the BSRTC classification (Table 5) [32–43, 45–59]. Italian studies based on the 2014 SIAPEC classification report an average malignancy rate of 14.2% for TIR3a nodules and 44.7% for the TIR3b cytological category in studies with adult cohorts [32–43]. In our cohort, we did not observe any malignancy among TIR3a nodules but the amount of histological data was very small, although the follow-up of 4.6 years confirmed the cytological category in all subjects who performed clinical follow-up with FNAB repeated every 12–18 months. The malignancy rate among subjects with TIR3b nodule was 77.8%, which is much higher than the rate observed in adults, similarly to other studies reporting pediatric cohorts [53]. The mean observed malignancy rate among pediatric studies using the BSRTC system is 39.5% for AUS/FLUS category and 41.5% for the SFN category, demonstrating greater sensitivity of the SIAPEC classification to sort out benign nodules and malignancies also in pediatric age. A recent meta-analysis based on the BSRTC system showed a lower ROM in adults with indeterminate nodules (30.5 and 28.9%, respectively) with no substantial differences between the two categories AUS/FLUS and SFN as reported in pediatric studies [59]; this finding is especially important when deciding on the surgical approach.

**Table 5:** Comparison of the present study, the main adult studies based on the SIAPEC classification and the main pediatric studies based on the BSRTC system

Reference	FNAB/Histology	TIR1	TIR2	TIR3a	TIR3b	TIR4	TIR5
Present study	75/40	0%	0%	0%	77.8%	100%	100%
Adult studies based on SIAPEC 2014							
Tartaglia [32]	-/52 (only indeterminate)	–	–	6.7%	54.5%	–	–
Medas [33]	-/102 (only indeterminate)	–	–	21.1%	57.8%	–	–
Ulisse [34]	-/50 (only indeterminate)	–	–	13.1%	44.4%	–	–
Straccia [35]	452/172 (only indeterminate)	–	–	16%	28%	85%	100%
Trimboli [36]	63/51 (only indeterminate)	–	–	7.5%	54.5%	–	–
Rullo [37]	-/290 (only indeterminate)	–	–	10.2%	43.8%	–	–
Valebrega [38]	157/75 (only indeterminate)			4%	48%		
Trimboli [39]	-/74 (only indeterminate)	–	–	12.1%	42.4%	–	–
Quaglino [40]	-/150 (only indeterminate)	–	–	20.8%	60.3%	–	–
Rullo [41]	-/111 (only indeterminate)			14.5%	54%		
Sparano [42]	562/273 (only indeterminate)	–	–	25%	40.4%	–	–
Cozzolino [43]	96/65 (only TIR3b)	–	–	–	38.5%	–	–
Total		–	–	**14.2%**	**44.7%**	–	–
Pediatric studies based on BSRTC							
Monaco [45]	179/96	0%	7%	28%	58%	100%	100%
Rossi [46]	245/64	0%	0%	11.8%	81.8%	100%	100%
Lale [47]	282/78	17%	0%	50%	47%	100%	100%
Norlen [48]	66/39	0%	0%	22%	100%	100%	100%
Buryk [49]	76/36	0%	10%	0%	50%	86%	100%
Amirazodi [50]	207/65	0%	16%	67%	–	71%	100%
Pantola [51]	218/44	0%	0%	8.3%	10%	100%	100%
Partyka [52]	186/61	0%	1.5%	21%	57.1%	100%	100%
Cherella [53]	430/190	11%	0.7%	44%	71%	73%	97%
Heider [54]	46/41	–	–	36%	20%	100%	–
Kardelen [55]	80/37	0%	25%	100%	75%	85.7%	100
Wang [56]	302/104	0%	7%	20%	25%	100%	100%
Suh [57]	141/111	22%	3.2%	75%	50%	100%	100%
Vuong [58]	827/300	30%	50%	66.6%	36.4%	100%	99.3%
Total		–	–	**39.5%**	**41.5%**	–	–

The bold values indicates the statistically significant results

ATA thyroid cancer guidelines for the pediatric age recommend surgery (lobectomy + isthmusectomy) for indeterminate FNAB categories in accordance with Bethesda classification. Considering that the 2014 SIAPEC classification seems to have a higher sensitivity to sort out benign and malignant nodules, the high ROM observed in the TIR3b group may orient toward clinical follow-up in the indeterminate TIR3a and total thyroidectomy in the TIR3b subcategory, especially in the case of bilateral or of right lobe localization. However, further evidence is needed from studies with larger cohort sizes to confirm the significant gap in the ROM between TIR3a and TIR3b in pediatric age.

The present study was conducted in a single tertiary pediatric endocrinology center over a period of 20 years. The strengths are the experience acquired by the center and the centralization of the cytological and histological data. These data were also compared with the main pediatric studies based on the BSRTC system and with the main Italian studies on adults based on the SIAPEC classification. Given the apparent greater sensitivity of this classification, the reported data should encourage Italian endocrinologists to differentiate benign and malignant nodules within the indeterminate category, to better orient clinicians toward clinical follow-up or surgery follow-up management.

The study also has many limitations. The data are retrospective and the follow-up period after 2014 is too short. The size of the cohort with indeterminate nodules is too small to have a strong statistical relevance of the data presented, but the rarity of thyroid nodule in pediatric age should be considered, as well as the fact that the new SIAPEC classification was available starting from 2014.

In conclusion, this is the first pediatric study that analyzes the performance of the SIAPEC cytological classification for thyroid nodules. The data reported seem to confirm the greater sensitivity of this classification in identifying benign and malignant nodules in the indeterminate category also in pediatric age and not only in adults. This finding, together with the fact that a higher ROM was observed in pediatric age, may orient clinicians toward a surgical approach using total thyroidectomy in the indeterminate TIR3b group, although this hypothesis should be *necessarily* confirmed in further national multicenter studies with a larger cohort size.

## Data Availability

All data can be available upon request to the corresponding Author.
